# Local thermal adaptation and limited gene flow constrain future climate responses of a marine ecosystem engineer

**DOI:** 10.1111/eva.12909

**Published:** 2020-01-25

**Authors:** Adam D. Miller, Melinda A. Coleman, Jennifer Clark, Rachael Cook, Zuraya Naga, Martina A. Doblin, Ary A. Hoffmann, Craig D. H. Sherman, Alecia Bellgrove

**Affiliations:** ^1^ School of Life and Environmental Sciences Centre for Integrative Ecology Deakin University Geelong Vic. Australia; ^2^ Deakin Genomics Centre Deakin University Geelong Vic. Australia; ^3^ NSW Fisheries National Marine Science Centre Coffs Harbour NSW Australia; ^4^ Climate Change Cluster University of Technology Sydney Sydney NSW Australia; ^5^ Department of Botany University of British Columbia Vancouver BC Canada; ^6^ School of BioSciences Bio21 Institute The University of Melbourne Parkville Vic. Australia

**Keywords:** climate change, evolutionary potential, gene flow, local adaptation, marine ecosystem engineer

## Abstract

Rising ocean temperatures and extreme temperature events have precipitated declines and local extinctions in many marine species globally, but patterns of loss are often uneven across species ranges for reasons that are poorly understood. Knowledge of the extent of local adaptation and gene flow may explain such patterns and help predict future trajectories under scenarios of climate change. We test the extent to which local differentiation in thermal tolerance is influenced by gene flow and local adaptation using a widely distributed intertidal seaweed (*Hormosira banksii*) from temperate Australia. Population surveys across ~2,000 km of the species range revealed strong genetic structuring at regional and local scales (global *F*_ST_ = 0.243) reflecting extremely limited gene flow, while common garden experiments (14‐day exposures to 15, 18, 21°C) revealed strong site differences in early development and mortality in response to elevated temperature. Embryos from many sites spanning a longitudinal thermal gradient showed suppressed development and increased mortality to elevated water temperatures, but populations originating from warmer and more variable thermal environments tended to be less susceptible to warming. Notably, there was significant local‐scale variation in the thermal responses of embryos within regions which was corroborated by the finding of small‐scale genetic differences. We expect the observed genetic and phenotypic differentiation to lead to uneven responses to warming sea surface temperatures in this important marine foundation species. The study highlights the challenges of predicting species responses to thermal stress and the importance of management strategies that incorporate evolutionary potential for “climate‐proofing” marine ecosystems.

## 1 INTRODUCTION

The speed and magnitude of biotic shifts being triggered by climate change pose a major challenge for marine biodiversity conservation. Rising ocean temperatures, increasing acidification, and changing ocean currents are contributing to fundamental and irreversible ecological transformations in marine ecosystems at a global scale (Babcock et al., 2019; Harris et al., 2018; Hoegh‐Guldberg & Bruno, 2010). Species living close to their physiological limits are of particular concern and will become increasingly dependent on their ability to overcome environmental change via dispersal, physical tolerance, and evolutionary adaptation (Hoffmann & Sgro, 2011; Portner & Gutt, 2016). Understanding the environmental resilience of ecosystem engineers (i.e., keystone and foundation species) is of particular importance, given their response to environmental change will have major impacts on community structure and ecosystem function (Babcock et al., 2019; Hoegh‐Guldberg & Bruno, 2010; Smale et al., 2019; Thomson et al., 2015). Such information is necessary to inform marine conservation planning directed at minimizing biodiversity loss, and preserving environmental, economic, and cultural values (Magris, Pressey, Weeks, & Ban, 2014).

Macrophytes are key foundation species in temperate oceans underpinning reef ecosystem services (Dayton, 1972; Jones, Lawton, & Shachak, 1994) but are at increasing threat from environmental change with declines seen as a result of warming (Vergés et al., 2016, 2014), heatwaves (Thomson et al., 2015; Wernberg, Bennett, et al., 2016), over‐harvesting (Steneck et al., 2002), and urbanization (Airoldi & Beck, 2007; Coleman, Kelaher, Steinberg, & Millar, 2008; Connell et al., 2008). Critically, such declines appear to be long term or even permanent, highlighting a need to understand the adaptability and vulnerability of marine macrophytes to future change. This is pertinent in areas where marine macrophyte communities are showing signs of acute and chronic climate stress, such as climate change “hot spots” that are particularly prone to extreme temperature events, and that are experiencing sea surface temperature orders of magnitude above the global average (Babcock et al., 2019; Hobday & Pecl, 2014; Wernberg et al., 2011). This is a major concern as thermal stress is known to impair photosynthetic, respiratory, and cellular function in macrophyte species, inhibiting growth, inducing mortality and disease, and resulting in range contractions and local extirpations (Flukes, Wright, & Johnson, 2015; Mathieson & Dawes, 1986; Pineiro‐Corbeira, Barreiro, Cremades, & Arenas, 2018; Smale & Wernberg, 2013; Wernberg, Bettignies, Joy, & Finnegan, 2016). Furthermore, thermal stress can suppress macrophyte resilience to environmental stressors associated with other anthropogenic and natural perturbations (Wernberg et al., 2010) as well as alter key ecological interactions that impact macrophyte populations, such as grazing and predation (Miranda et al., 2019; Provost et al., 2017; Vergés et al., 2014). Coupled climate–ecosystem models suggest that declines are likely to intensify in coming decades, with projections of major range contractions of temperate seaweeds, and potential risks of extinction for many species (Martinez et al., 2018).

The resilience of marine macrophyte populations to environmental change will be largely determined by levels of standing adaptive genetic variation and patterns of gene flow among populations. Traditionally, insights into climate resilience have been gained by characterizing patterns of gene flow among populations distributed across thermal gradients. While gene flow can be an impediment to local adaptation, it can also assist the adaptation process by making thermally adapted genotypes available for selection (Hoffmann & Sgro, 2011; Hoffmann & Willi, 2008). Also, quantitative approaches (e.g., common garden and transplant experiments) are used to assess the extent to which species and populations have adapted to different environments, by testing the genetic basis of trait variation spanning environmental gradients (Hoffmann & Willi, 2008). Such studies have been particularly useful for assessing historical responses to environmental change and predicting the evolvability of species from both marine and terrestrial systems (Sgro, Lowe, & Hoffmann, 2011; Sherman & Ayre, 2008), but they are limited for marine macrophytes.

Predicting species responses to warming environments requires an understanding of intraspecific variation in thermal responses and gene flow across species ranges (King, McKeown, Smale, & Moore, 2018). Predictive tools such as species distribution and climate niche models do not directly consider physiological variation unless this variation contributes to extant distributions (Razgour et al., 2019; Willis et al., 2015). Such models may therefore be limited in predicting range shifts, particularly at edges of a species’ distribution, where temperatures are expected to exceed theoretical species “thermal niche” limits or safety margins (Bennett, Wernberg, Joy, Bettignies, & Campbell, 2015), which may be countered by evolutionary shifts (Bush et al., 2016). Variability in thermal physiology between populations has been established for several marine macrophytes (reviewed in King et al., 2018), which may contribute to uneven population responses to thermal stress (Bennett et al., 2015; Carballo, Olabarria, & Osuna, 2002; Filbee‐Dexter, Feehan, & Scheibling, 2016; Starko et al., 2019; Tegner & Dayton, 1987; Thomsen et al., 2019). This variation often coincides with fine‐scale genetic structuring (King et al., 2018), but empirical tests of this are lacking. Information on the genetic structure of populations and phenotypic differences among populations is needed to help understand the uneven thermal response of populations, identify those most vulnerable to thermal stress, and indicate where genotypes resilient to future climate changes might reside. These are all important components of climate change adaptation and restoration programs (Foden et al., 2019; Jordan, Hoffmann, Dillon, & Prober, 2017; Willis et al., 2015; Wood et al., 2019).

*Hormosira banksii* (Turner) Decaisne is the dominant intertidal macrophyte across temperate Australasia and is an autogenic ecosystem engineer with no functional equivalents (Bishop et al., 2009; Pocklington, Keough, O'Hara, & Bellgrove, 2019; Schiel, 2006). This species is highly sensitive to environmental disturbance associated with coastal development and urbanization (Doblin & Clayton, 1995; Keough & Quinn, 1998; Kevekordes, 2000), and thermal stress (Alestra & Schiel, 2015; Tait & Schiel, 2013). *H. banksii* embryo development is particularly sensitive to upwards shifts in temperature, with water 3°C higher than ambient levels causing significant mortality (Alestra & Schiel, 2015; Clark, Poore, Ralph, & Doblin, 2013). Yet significant genotype × environment interactions for embryo growth have been reported, as well as growth and photosystem traits that show heritable genetic variation relating to temperature (Clark, Poore, & Doblin, 2018; Clark et al., 2013). These findings suggest there is potential for selection to result in the evolution of more thermally tolerant populations. However, despite potential standing genetic variation in some populations for thermal adaptation, gene flow may be limited in *H. banksii,* restricting the adaptive potential of this species. Previous studies indicate strong genetic structuring among populations from the central region of Australia's east coast (Coleman et al., 2011; Coleman, Clark, Doblin, Bishop, & Kelaher, 2019) suggesting that gene flow is unlikely to facilitate the natural movement of genetic variants across temperature gradients to aid adaptation and recovery of depleted populations. Such genetic structuring could also contribute to local adaptation and variation in thermal tolerances among populations spanning thermal gradients in Australian temperate waters. However, the genetic structure across the full range of this species, and levels of population variability in thermal tolerance, is currently unknown.

In this study, we assessed the potential adaptability of *H. banksii* to rising ocean temperatures across much of its distribution. First, we use microsatellite markers to investigate patterns of genetic diversity, gene flow, and population connectivity from sites spanning ~2,000 km of the distribution of this species around mainland Australia and covering a wide temperature gradient (mean summer sea surface temperatures ranging from 16 to 24°C). Second, we undertake a common garden experiment to explore spatial variation in thermal responses among populations with different thermal histories by comparing embryo development at different experimental temperatures. Outcomes from these analyses will help determine the role of gene flow and local adaptation in affecting responses of populations to thermal stress. We expected populations under thermal extremes in the sampling range to be phenotypically adapted to those temperature environments, particularly in the presence of low levels of gene flow. Any adaptive changes would produce uneven thermal responses among populations across the species range. We discuss the implications of our results in the context of the future evolutionary potential of *H. banksii*, and conservation/ restoration approaches that might be considered to catalyze adaptation and recovery processes in light of climate change.

## 2 METHODS

### 2.1 Study species

*Hormosira banksii* is a perennial, dioecious, habitat‐forming fucoid macroalga with a distribution extending >2,000 km from Albany in Western Australia to Lennox Head in New South Wales on mainland Australia, around Tasmania, the North and South Islands of New Zealand, and some of the smaller offshore islands in southern Australasia (Clark et al., 2018; Osborn, 1948). *H. banksii* is fertile throughout the year, releasing gametes on emersion during low tide (Levring, 1949). Once fertilized, eggs are thought to attach rapidly, thereby limiting dispersal (Bellgrove, Clayton, & Quinn, 1997, 2004). The thallus of *H. banksii* consists of multiple chains of hollow, water‐filled, vesicles that arise from a diffuse holdfast (Osborn, 1948) capable of vegetative regeneration (Keough & Quinn, 1998; Schiel & Taylor, 1999), with individual plants surviving >11 years (Kain, 2015). *H. banksii* shows significant morphological variability among populations and across geographic regions, and between marine and estuarine environments shown to have functional consequences (Clark et al., 2018). This is thought to be correlated with local environmental conditions (Macinnis‐Ng, Morrison, & Ralph, 2005; Ralph, Morrison, & Addison, 1998) and may be linked to genetic differentiation (Coleman et al., 2019).

### 2.2 Population genetic analysis

#### 2.2.1 Sampling

**Figure 1:**
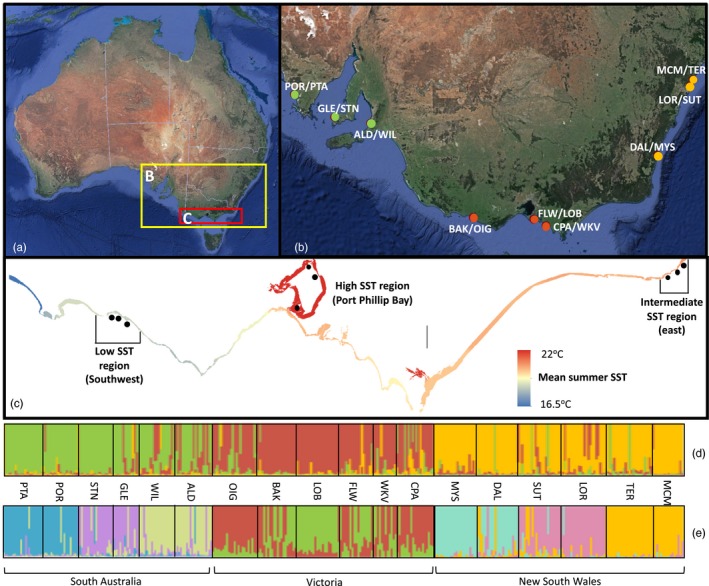
Maps showing *Hormosira banksii* sampling locations for the population genetic analysis (map “B”) and common garden experiments (map “C,” modified from Ierodiaconou et al., 2018). Map c shows the nesting of collection sites within three distinctive temperature zones (low, intermediate, and high), with the coastline color coded according to local mean summer sea surface temperatures (SST). Summary plots of the population subdivision based on STRUCTURE analyses of *Hormosira banksii* multilocus genotypes are also included (“d” and “e”). Single vertical lines broken into segments reflect each individual in the STRUCTURE summary plot, where segments are proportional to the membership coefficient for each of the population clusters. Individuals are arranged into sites from which they were sampled following the order given in Table 1 (site codes are also derived from Table 1). STRUCTURE plots are provided for the first hierarchical level of structure (all states included in the analysis; plot “d”) and the second hierarchical level of structure (independent analysis of sites from New South Wales, Victoria, and South Australia; plot “e”)

Tissue samples of *H. banksii* were collected between December 2006 and April 2007 at 3 spatial scales across the geographic distribution of this species, with nesting present within each scale: large scale (1,000s km separation between states and across the Tasman Sea to New Zealand; hereafter referred to as *states*), intermediate scale (*regions* separated by 100s km), and small scale (*sites* separated by 10s km). We sampled from two rocky‐shore *sites* within each of nine *regions* within three *states* (a total of 18 sample locations): three regions in each of South Australia, Victoria, and New South Wales (Table 1; Figure 1a,b). At each site, 1–2 vesicles from each of 32 individual clumps (defined as an individual with one holdfast and multiple fronds) of *H. banksii* were collected, washed in freshwater, blotted dry, and desiccated in silica gel for later genetic analysis. Genomic DNA was extracted from 10 mg sample of dried material tissue for individual specimens using the NucleoSpin® 96 Plant II protocol (Macherey‐Nagel Inc., Düren, KO, GER).

**Table 1:** Location details and corresponding codes for 18 collection sites of *Hormosira banksii* from southern, southeastern, Western Australia, and New Zealand which were used for genetic analyses

Region	Site	Code	Latitude	Longitude
New South Wales				
Central Coast	McMasters	MCM	−33.5011	151.4261
	Terrigal	TER	−33.4565	151.4385
Sydney Region	Long Reef	LOR	−33.7378	151.3131
	Sutherlands Point	SUT	−34.0035	151.2282
South Coast	Dalmeny	DAL	−36.1628	150.1297
	Mystery Bay	MYS	−36.3036	150.1389
Victoria				
Wilsons Promontory	Cape Patterson	CPA	−38.6667	145.6167
	Walkerville	WKV	−38.8333	146.0000
Mornington Peninsula	Flinders West	FLW	−38.4667	145.1000
	London Bridge	LOB	−38.3167	144.6833
Southwest Coast	Backyards	BAK	−38.4333	142.5833
	Oigles	OIG	−38.3833	142.2167
South Australia				
Fleurieu Peninsula	Aldinga	ALD	−35.2833	138.4403
	Willunga	WIL	−35.2703	138.4431
Yorke Peninsula	Gleeson Landing	GLE	−35.0089	136.9506
	Stenhouse	STN	−35.2794	136.9422
Eyre Peninsula	Porter Bay	POR	−34.1278	135.2664
	Point Avoid	PTA	−34.6781	135.3267

#### 2.2.2 Microsatellite analysis

A total of 373 *H. banksii* individuals from 26 sites spanning New South Wales, Victoria, and South Australia were genotyped at 10 microsatellite loci previously developed by Bellgrove et al. (2017). Loci were amplified by multiplex PCR using Eppendorf Mastercycler *S* gradient units following the protocol described by Blacket, Robin, Good, Lee, and Miller (2012). Genotyping was performed using an Applied Biosystems 3730 capillary analyzer (AGRF, Melbourne Australia), and microsatellite profiles were examined and scored manually using GENEMAPPER version 4.0 (Applied Biosystems). Independence of loci (absence of linkage) was assessed using the software GENEPOP, version 3.4 (Raymond & Rousset, 1995) and the probability test function, which found no significant association among loci. The software MICRO‐CHECKER (Van Oosterhout, Hutchinson, Wills, & Shipley, 2004) was used to assess microsatellite loci for null alleles and scoring errors using formula 1 outlined by Brookfield (1996) and found no evidence of null alleles across sites and loci. Descriptive statistics were calculated for the microsatellite data using FSTAT version 2.9.3 (Goudet, 1995), including allelic richness per population averaged over loci, Weir and Cockerham's inbreeding coefficient (*F*_IS_: the deficiency of heterozygotes relative to the level expected with random mating), and a global estimate of population differentiation (*F*_ST_) with 95% confidence limits (Weir & Cockerham, 1984). In addition, population pairwise measures of *F*_ST_ and their significance were determined using permutation (10,000 permutations). In order to overcome potential limitations of *F*_ST_ calculations using multiallelic loci (Jost, 2008), additional estimates of population differentiation, global *D*_est_, and population pairwise measures of *D*_est_ (significance determined using 10,000 permutations) were generated using GenAlEx 6.5 (Peakall & Smouse, 2006). The false discovery rate (FDR) procedure (Benjamini & Hochberg, 1995) was used to adjust significance levels when performing multiple simultaneous comparisons.

Estimates of observed (Ho) and expected (He) heterozygosity were determined using the Excel Microsatellite Toolkit (Park, 2001) and deviations from Hardy–Weinberg equilibrium (HWE) were determined using Genepop version 3.4 (Raymond & Rousset, 1995). An analysis of molecular variation (AMOVA) was performed in GenAlEx 6.5 (Peakall & Smouse, 2006) using pairwise *F*_ST_ as the distance measure, with 10 000 permutations and missing data for loci set at 10%. The model for analysis partitioned variation among regions, among sample sites within regions, and within sample sites. A discriminant analysis of principal components (DAPC), implemented in the adegenet package for *R* (Jombart, 2008; Jombart & Ahmed, 2011), summarized patterns of genetic differentiation between sample sites.

Bayesian analyses implemented in STRUCTURE (Pritchard, Stephens, & Donnelly, 2000) were conducted to estimate the number of populations within the sample data. STRUCTURE identifies the number of distinct population clusters, assigns individuals to clusters, and identifies migrants and admixed individuals using genetic data only. To determine the number of populations (*K*), five independent simulations for *K* = 1–18 with 100,000 burn‐in and 1,000,000 data iterations were run. Analyses were performed using the admixture model of population structure (i.e., each individual draws some fraction of their genome from each of *K* populations) with no prior population information included, and allele frequencies set as independent among populations. The most likely *K* was estimated using Evanno's Δ*K* (Evanno, Regnaut, & Goudet, 2005) in Structure Harvester (Earl & Vonholdt, 2012).

Rates of recent migration were estimated among each of the sampling locations using a Bayesian algorithm implemented in BAYESASS v.3.0.3 (Wilson & Rannala, 2003). The program estimates migration among populations within the last three generations. To identify movements among populations, five independent runs of 10^7^ Markov chain Monte Carlo (MCMC) iterations were used following a burn‐in period of 10^7^ and a sampling interval of 500 steps. Chains were compared with a stationary posterior distribution for convergence by performing multiple runs with dispersed starting values. The proportion of individuals that were assigned as migrants (migration rates), and associated 95% confidence intervals (CIs) were estimated among each of the sampling locations.

### 2.3 Testing for local adaptation

#### 2.3.1 Site selection and sampling

We tested for genetically based variation in thermal responses by performing common garden experiments on *H. banksii* sampled from across a longitudinal temperature gradient in south eastern Australia. Sampling was conducted according to a spatially hierarchical design, with *H. banksii* sampled from three sites nested within three distinct temperature regions of the Victorian coastline (Table 2; Figure 1c). Processed sea surface temperature (SST) datasets for the years 1995–2015 were evaluated from the Integrated Marine Observing System (IMOS; Ierodiaconou et al., 2018) and used to identify the three most variable SST zones, including southwestern Victoria (low temperature and variability), eastern Victoria/southern NSW (intermediate temperature and variability), and Port Phillip Bay (high temperature and variability) (hereafter Southwest, East, and PPB, respectively; Figure 1c). Sea surface temperature rather than air temperature was recognized as the most important variable for early life stage development, as fertilization and early embryonic development occurs during submersion after gamete release during low tide (Bellgrove, McKenzie, McKenzie, & Sfiligoj, 2010), and intertidal seaweeds are most physiologically competent during periods of submersion (Hurd, Harrison, Bischof, & Lobban, 2014). Sampling was performed in October 2018 (austral spring), with simultaneous collections from all three regions. Approximately 2 kg of mature *H. banksii* fronds was randomly collected by hand at low tide from a minimum of 50 individuals distributed over an area of 50–100 m^2^ at each site. Samples were transported to the laboratory on ice and stored in labeled plastic bags at 4°C in the dark for less than 3 days prior to experimentation. Sites from the southwestern and eastern Victorian regions were included in both the population genetic and common garden experiments, while sites from Port Phillip Bay were used only for the common garden experiment.

**Table 2:** Location details for 9 collection sites of *Hormosira banksii* from southeastern Australia used for the common garden experiment, including mean summer and mean seasonal ranges in sea surface temperature in decimal degrees (**°**C)

Region	Location/Site	Latitude	Longitude	Sea surface temperature (°C)	
				Mean summer	Mean seasonal range
Southwest	Warrnambool	−38.4044	142.4760	18.12	4.11
	Backyards	−38.4384	142.5877	18.04	3.89
	Peterborough	−38.6061	142.8696	17.86	3.84
Port Phillip Bay	Queenscliff	−38.2779	144.6401	19.91	7.04
	Williamstown	−37.8694	144.8901	21.57	9.92
	Black Rock/Half Moon Bay	−37.9942	145.0509	21.61	9.85
East	Mallacoota	−37.5684	149.7644	19.08	4.85
	Merimbula	−36.8997	149.9152	20.33	4.81
	Eden	−37.0567	149.9131	20.28	4.80

#### 2.3.2 Thermal response experiments

*H. banksii* zygotes from each of the three defined thermo‐geographic regions (Southwest, East, and PPB) were reared under common culture conditions for experimental purposes following the collections outlined in Section 2.3.1. Thermal response experiments were performed by exposing zygotes from each site to three controlled temperature treatments, 15, 18, and 21℃. These temperatures were based on 15 and 18℃ falling within the bounds of annual sea surface temperatures (SST) across all regions, while only sites from the PPB region are exposed to average summer SSTs as high as 21℃ (Table 2). Initially, gamete release for all sample sites was stimulated in accordance with Kevekordes and Clayton (1996), whereby thalli were rinsed in freshwater, patted dry using paper towel, and exposed to ambient temperature and light for up to 1 hr until gametes were exuded. Eggs were collected prior to sperm to maximize fertilization success, as sperm viability is known to deteriorate quickly upon release into seawater (Doblin & Clayton, 1995; Levring, 1949). Male and female gamete solutions were made for each of the nine sample locations by submerging 10 male or 10 female fronds in 150 ml of 15℃, 0.22 µm filtered seawater (Sterivac Millipore Corporation, Bedford MA). Relative gamete concentrations were determined using UV spectrophotometry and subsequently standardized using 15℃ microfiltered seawater to achieve female:male gamete concentrations of approximately 1:50 to avoid polyspermy (Kevekordes & Clayton, 1996). Once standardized, gamete solutions from each source population were mixed to initiate fertilization (within population crosses only), with 700 µl from each zygote solution immediately pipetted onto 6 individual glass coverslips placed inside each of 18 replicate Petri dishes (6 for each temperature treatment) and left to settle for approximately 5 min (Kevekordes & Clayton, 1996). Twenty mL of 15℃ microfiltered seawater was gently added to each Petri dish to submerge the glass coverslips, after which six Petri dishes from each sample location were randomly assigned to one of three temperature‐control cabinets set at 15, 18, and 21℃, fitted with full spectrum (T5 Fluorescent Kit 4 × 54 Watt Hydro 44, 35 μmolm^‐2^s^‐1^) lights on 12:12‐hr light:dark cycle. Petri dishes representing the different sample locations were also randomized within the cabinets.

Embryonic development for each sample location under each temperature treatment was assessed microscopically (100× magnification, ZEISS—Axiostar Plus Microscope), with developmental milestones being recorded for 100 individuals per replicate, adapted from Kevekordes and Clayton (1996): fertilization at 1–2 hr, >4 cell divisions and rhizoid length at 7 days, and mortality and meristematic activity (evidenced by presence of apical hairs) at 14 days. Fertilization success was scored from one randomly selected replicate glass coverslip in each of three replicate Petri dishes for each sample location and temperature treatment prior to manipulation, whereas all other dependent variables were scored from one randomly selected replicate glass coverslip from each of the six replicate Petri dishes for each sample location and temperature treatment. If insufficient zygotes had settled onto a single coverslip for scoring, a second one was sampled. At day 7, photographs of embryos were taken with a mounted camera (ZEISS—Axiocam ERc 5s) at ×100 magnification and subsequently 5 embryos randomly selected from each replicate for measurement of the rhizoid lengths using the ImageJ analysis package (version: 2.0.0‐rc‐43/1.51p, https://imagej.net).

#### 2.3.3 Statistical analyses for common garden experiment

Fertilization success prior to temperature manipulation was analyzed by a two‐factor nested analysis of variance (ANOVA) with *region* (three levels: Southwest, East, and PPB; fixed) and *sites nested within regions* (nine levels; random). Data from the common garden experiment were then analyzed by partially nested, mixed model (split plot) design ANOVAs with *temperature* (three levels: 15, 18, and 21°C; fixed), *region* (three levels: Southwest, East, and PPB; fixed), and *sites nested within regions* (nine levels; random). Assumptions of normality and homogeneity of variances were visually checked with box plots and residual plots (Quinn & Keough, 2002). Where significant *temperature* × *region* interactions occurred, simple main effects (*SEM*) analyses examined the effects of temperature for each region separately with a reduced 2‐factor ANOVA design (*temperature* and *site*) using the error term from the full model to test the effect of temperature (Quinn & Keough, 2002). Tukey's HSD pairwise comparisons compared temperature treatments after ANOVAs. All analyses were performed on untransformed data in SYSTAT v13.2 and tested at *α* = 0.05.

Relationships between home site temperature environments and thermal response were estimated by regressing site‐level mean summer sea surface temperatures and mean seasonal variation in sea surface temperatures (evaluated from the Integrated Marine Observing System (IMOS) for the years 1995–2015) against mean values for each thermal response variable from each site in the 21°C treatment of the common garden experiment.

*Q*_ST_–*F*_ST_ comparisons were also performed to determine whether the degree of phenotypic differentiation observed among sites departs from neutral expectations, where *Q*_ST_ > *F*_ST_ suggests that quantitative traits show a higher level of differentiation than what would have been expected under the influence of genetic drift, indicative of selection. The *R* package Pstat (Da Silva & Da Silva, 2018) was used to estimate *P_ST_* (analogous to *Q*_ST_) for each thermal response variable for each site in the 21°C treatment (the highest experimental temperature), using *P*_ST_ = *c/h* ^2^σ^2^ *_b_*/(*c/h* ^2^σ^2^ *_b_* + 2σ^2^ *_w_*), where σ^2^ *_b_* and σ^2^ *w* are the respective phenotypic variances between and within groups of populations, *c* is an estimate of the proportion of the total variance due to additive genetic effects across populations, and *h* ^2^ is narrow‐sense heritability, the proportion of phenotypic variance due to additive genetic effects (Brommer, 2011). The *c/h* ^2^ default level of 1 was used; 95% confidence intervals were calculated with 1,000 bootstrapping data frames. As values of *c/h* ^2^ are often not known for wild populations and can be variable among populations, the robustness of this ratio on phenotypic divergence was evaluated using additional *c/h*_2_ values of 0.5 and 0.1. As microsatellite genotypes were not available for sites included in the common garden experiment, we contrasted *P*_ST_ against global *F*_ST_, and against estimates of genetic divergence between and within regions.

## 3 RESULTS

### 3.1 Population genetic analysis

Across the 10 microsatellite loci and 18 sample locations, we detected a total of fifty‐one alleles (one of which was exclusive to South Australian sites), with a mean of 6.0 alleles per locus (range 4–8). Levels of genetic diversity (allelic richness and heterozygosity) were largely consistent across sites over all loci and heterozygosity, with a mean allelic richness of 2.59 (range 2.16–3.00) and a mean observed heterozygosity of 0.41 (range 0.31–0.48; Table S1). Most sites were found to conform to HWE (Table S1), with the exceptions of sites FLW, LOB, and POR which all showed heterozygote deficits. This resulted in large and significant positive inbreeding coefficients (*F*_IS_) for these sites. Closer inspection indicated that a single locus influenced these deviations for site LOB, while several different loci influenced deviations for sites FLW and POR, suggesting caution when interpreting estimates of differentiation involving these sites.

We detected strong genetic structure among sites with global estimates of *F*_ST_ and *D*_est_ across all loci being significantly different from zero [*F*_ST_ = 0.243; 95% confidence interval (CI) = 0.20–0.29; *D*_est_ = 0.29; 95% CI = 0.17–0.31] indicating limited gene flow between sampling sites. Out of 153 pairwise population comparisons of *F*_ST_, only two did not differ significantly from zero, reflecting restricted gene flow among regions and most sites within regions (Table 3). Similarly, AMOVA analyses indicated that a significant proportion of the genetic variance (9%) could be attributed to difference among regions (*p* < .01, *F*_ST_ = 0.092), and 16% of the variance due to differences among sites within regions (*p* < .01, *F*_ST_ = 0.166). The relationship between genetic and geographic distance (Figure S1) suggested moderate isolation by distance (Mantel *r* = .35, *p* < .05). These analyses were repeated for New South Wales (NSW), Victoria (VIC), and South Australia (SA) separately, with only Victorian sites exhibiting significant isolation by distance (NSW: *r* = .07, *p* > .05; VIC: *r* = .35, *p* < .05; SA: *r* = .265, *p* > .05).

**Table 3:** Pairwise estimates of *F*_ST_ (lower diagonal) and *D*_est_ (upper diagonal) between 26 *Hormosira banksii* collection sites

	MCM	TER	LOR	SUT	DAL	MYS	CPA	WKV	FLW	LOB	BAK	OIG	ALD	WIL	GLE	STN	POR	PTA
MCM		0.14	0.24	0.23	0.12	0.30	0.25	0.25	0.28	0.39	0.41	0.39	0.26	0.23	0.35	0.29	0.37	0.23
TER	0.23		0.33	0.17	0.16	0.19	0.21	0.23	0.21	0.28	0.28	0.28	0.15	0.12	0.24	0.23	0.28	0.21
LOR	0.29	0.31		0.18	0.21	0.37	0.20	0.28	0.22	0.33	0.35	0.37	0.31	0.29	0.40	0.37	0.33	0.24
SUT	0.34	0.35	0.31		0.17	0.26	0.15	0.27	0.21	0.42	0.34	0.26	0.28	0.24	0.35	0.27	0.27	0.15
DAL	0.17	0.19	0.17	0.29		0.12	0.17	0.20	0.21	0.36	0.35	0.31	0.20	0.17	0.26	0.38	0.26	0.26
MYS	0.35	0.25	0.31	0.39	0.12		0.17	0.15	0.18	0.27	0.23	0.21	0.20	0.20	0.26	0.39	0.37	0.28
CPA	0.22	0.25	0.23	0.22	0.20	0.24		0.02	0.04	0.20	0.10	0.08	0.07	0.07	0.20	0.21	0.23	0.25
WKV	0.27	0.28	0.28	0.33	0.21	0.23	0.06		**0.03**	0.13	0.09	0.10	0.10	0.11	0.25	0.26	0.33	0.28
FLW	0.26	0.26	0.25	0.25	0.22	0.25	**0.03**	0.06		0.06	0.07	0.08	0.08	0.10	0.28	0.30	0.26	0.27
LOB	0.30	0.27	0.27	0.36	0.27	0.27	0.16	0.12	0.06		0.08	0.20	0.17	0.20	0.37	0.39	0.42	0.42
BAK	0.36	0.31	0.31	0.37	0.28	0.26	0.13	0.13	0.10	0.08		0.08	0.11	0.12	0.29	0.32	0.39	0.41
OIG	0.40	0.34	0.31	0.36	0.26	0.25	0.17	0.19	0.17	0.22	0.12		0.13	0.14	0.31	0.25	0.35	0.39
ALD	0.24	0.20	0.25	0.30	0.17	0.22	0.10	0.12	0.10	0.13	0.10	0.14		0.00	0.11	0.25	0.15	0.34
WIL	0.23	0.18	0.23	0.29	0.15	0.20	0.11	0.14	0.13	0.16	0.12	0.14	**0.00**		0.12	0.25	0.16	0.31
GLE	0.33	0.32	0.34	0.35	0.24	0.30	0.18	0.25	0.24	0.28	0.26	0.29	0.11	0.13		0.33	0.11	0.34
STN	0.32	0.28	0.32	0.33	0.31	0.37	0.21	0.27	0.27	0.30	0.28	0.26	0.22	0.22	0.29		0.43	0.27
POR	0.36	0.34	0.34	0.30	0.29	0.39	0.21	0.30	0.24	0.33	0.36	0.37	0.20	0.21	0.17	0.37		0.36
PTA	0.27	0.30	0.30	0.27	0.27	0.34	0.24	0.28	0.25	0.31	0.33	0.37	0.26	0.26	0.30	0.29	0.34	

Note

Values shown in bold are nonsignificant (*p* > .001) after 10,000 permutations and correction for multiple comparisons. Site codes are derived from Table 1.

Genetic differentiation between regions and sites within regions is depicted by the discriminant analysis of principal components (DAPC) of the microsatellite variation (Figure S2). When all sites were included in the analysis, clear regional differences were evident, with sites from NSW, VIC, and SA separating across the axes and representing three distinct clusters (estimated using the find cluster function; Figure S2a). DAPCs performed on sites from each state show regional genetic structuring with sites from common regions typically clustering more closely, but with centroids of population clusters rarely overlapping (Figure S2a–d). STRUCTURE Bayesian clustering analyses confirmed significant genetic differentiation between the NSW, VIC, and SA sites, with analyses indicating assignment of individuals from the respective states to three distinct population clusters (Δ*K* = 3; Figure 1d). STRUCTURE analysis of each region suggested significant structuring with each of the regions, consistent with the DAPC analysis. Further structuring at the state scale was evident when STRUCTURE analyses were performed on sites from each region independently (Figure 1e). Analyses of NSW sample locations revealed three distinct population clusters (Δ*K* = 3) corresponding with the Central Coast, Sydney, and South Coast regions. Similarly, significant regional differences were observed in SA where individuals were assigned to three population clusters (Δ*K* = 3) corresponding with the Fleurieu Peninsula, Yorke Peninsula, and Eyre Peninsula regions. Regional genetic structuring was less evident in Victoria where two population clusters were determined (Δ*K* = 2), but not reflecting an obvious geographic pattern. We expect this is likely due to comparatively lower level of genetic differentiation among Victorian sample locations, and the limited ability of STRUCTURE to resolve fine‐scale patterns of genetic structure (Evanno et al., 2005).

Results from the Bayesian migration analyses indicated limited migration among sites within the last three generations (Table S1). Estimates of the strength and directionality of migration demonstrated that each site has been largely dependent on recruitment from local sources, with minimal migration from nonlocal sources. Evidence of weak recent migration (average 8.3% migrants per generation) was recorded between three pairs of neighboring sites. In the Sydney region, evidence of unidirectional migration of 5% was found between LOR and SUT, while bidirectional migration of 9%–11% between sites WIL and ALD on the Fleurieu Peninsula separated by <2 km was also detected.

### 3.2 Testing for local adaptation

#### 3.2.1 Fertilization success

Fertilization success was high for all sites (mean ± *SE*: 92.7 ± 0.98%, *N* = 81 pooling across sites; Figure S3), and there was a marginally nonsignificant difference in fertilization success among regions (2‐factor nested ANOVA: *F*_(2,6)_ = 5.08; *p* = .051) and no significant difference among sites within regions (*F*_(6,72)_ = 1.09; *p* = .377) at the beginning of the experiment. This was expected as all gamete mixes were initially prepared under common temperatures (15°C), where fertilization was expected to be largely complete within minutes (Kevekordes & Clayton, 1996), prior to exposure to experimental temperatures.

#### 3.2.2 Embryo development and rhizoid growth (Day 7)

**Figure 2:**
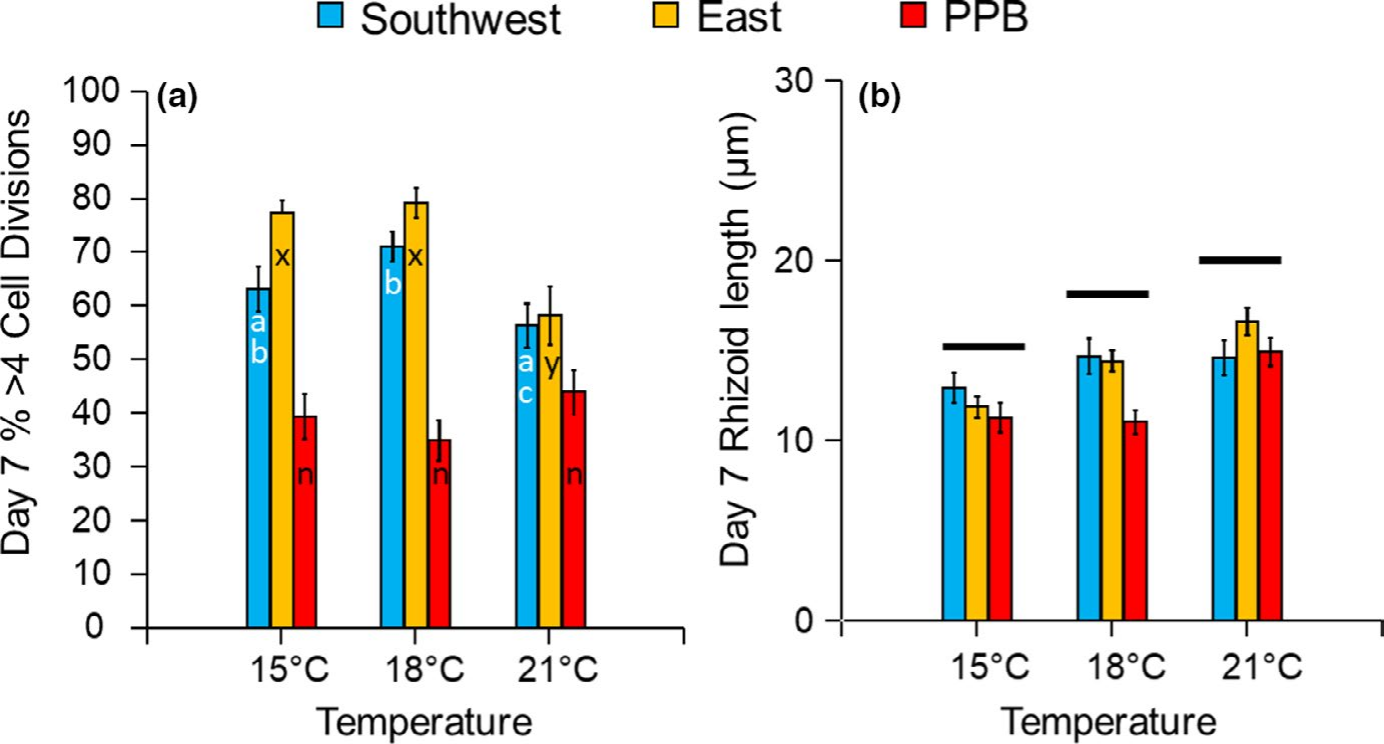
Development of embryos of *Hormosira banksii* from three source regions (southwest Victoria, eastern Victoria/southern NSW, and Port Phillip Bay, pooling three sites in each region) after 7 days of culture at 15, 18, and 21°C showing a) mean (±*SE*) percentage of embryos with greater than four cell divisions and well‐developed rhizoids, and (b) mean (±*SE*) rhizoid length. Broken horizontal lines above bars in (b) indicate significant differences among temperature treatments based on Tukey's test after the full ANOVA model, whereas letters on bars in (a) indicate Tukey's test results among treatments for each region individually after simple main effects analysis (because of significant *temperature* × *region* interaction in the full model)

The response of developing embryos to different temperature treatments was complex and variable among sites and regions. The percentage of mature embryos at day 7 (>4 cell divisions and presence of rhizoids) among temperature treatments were consistent across sites within regions (ANOVA *temperature × site(region)*; *F*_(12,135)_ = 0.77; *p* = .685) but not among regions (ANOVA *temperature × region*; *F*_(4,12)_ = 8.58; *p* = .002). There was no significant difference in the percentage of mature embryos among temperature treatments for sites from the PPB region (SME *temperature F*_(2,12)_ = 2.352; *p* = .137; Figure 2a), although this region did have the lowest proportion of mature embryos compared with sites from the Southwest and East regions. In contrast, the percentage of mature embryos differed between temperatures for the Southwest and East regions, with significantly lower development in the warmest treatment compared with 15 and 18°C (Tukey's HSD, *p* < .05; Figure 2a). Rhizoid lengths increased significantly with temperature, with length being greatest at 21°C (ANOVA *temperature*; *F*_(2,4)_ = 13.91; *p* = .001; Tukey's HSD *p* < .05 for all pairwise comparisons); this pattern was consistent across sites within regions (ANOVA *temperature* × *site(region)*; *F*_(12,134)_ = 1.66; *p* = .082) and among regions (ANOVA *temperature* × *region* interaction; *F*_(4,12)_ = 2.35; *p* = .114; Figure 2b).

#### 3.2.3 Embryo mortality and meristematic activity (Day 14)

**Figure 3:**
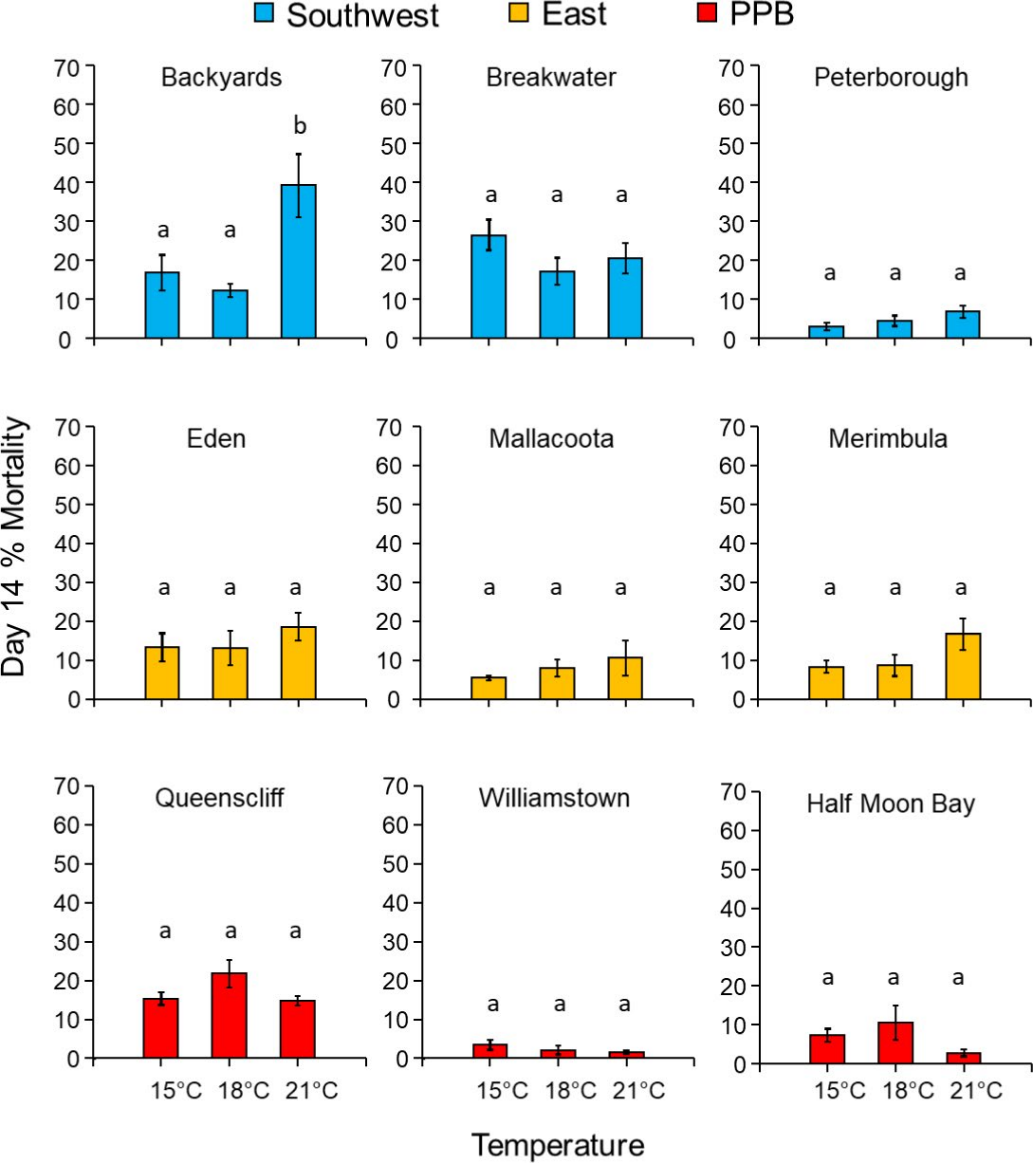
Mean (±*SE*) percentage mortality of embryos of *Hormosira banksii* from three random sites in each of three source regions (southwest Victoria, eastern Victoria/southern NSW, and Port Phillip Bay) after 14 days of culture at 15, 18, and 21°C. Letters above bars indicate Tukey's test results among temperature treatments for each site individually after simple main effects analysis (because of significant *temperature* × *site(region)* interaction in the full model)

**Figure 4:**
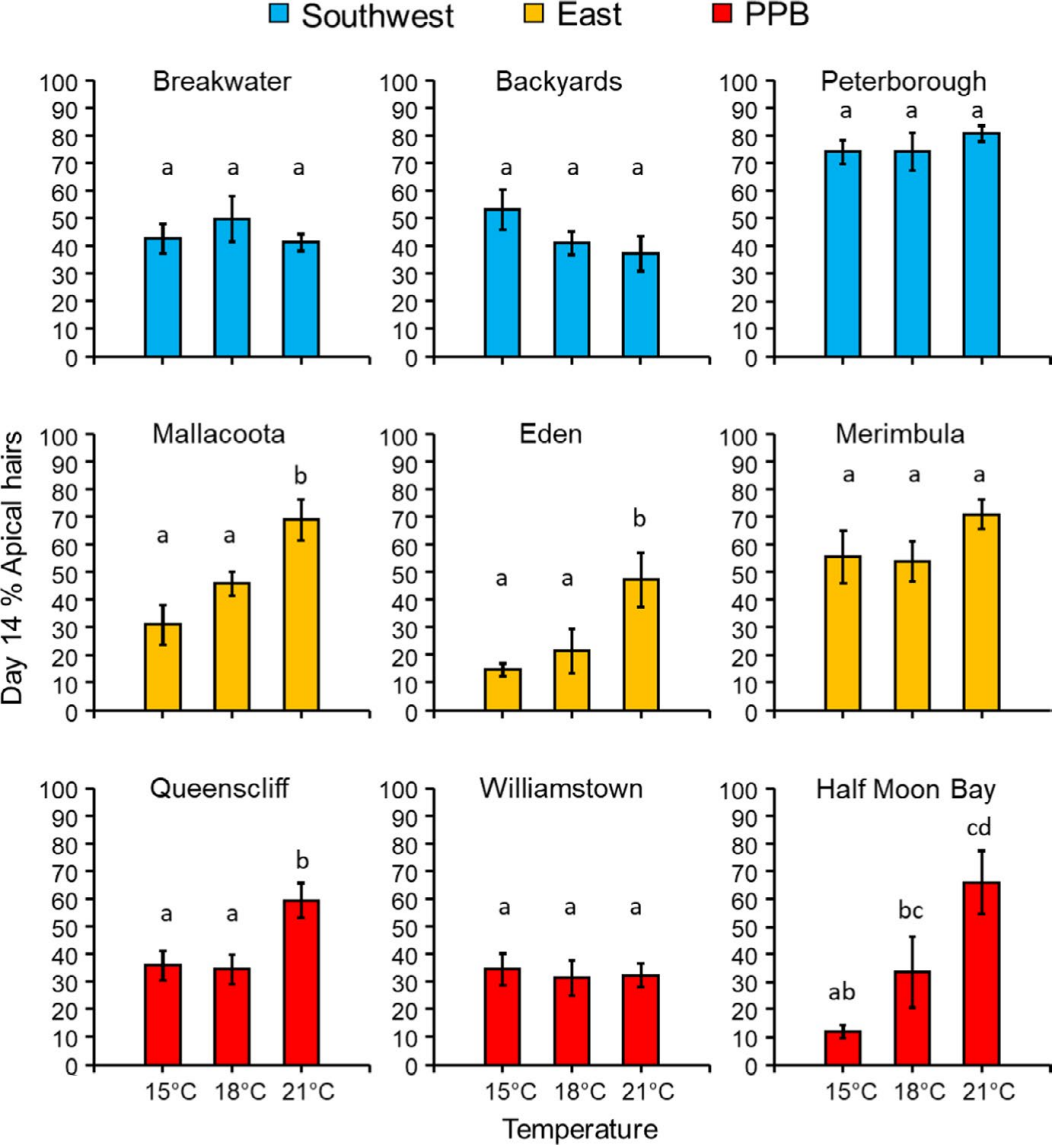
Development of embryos of *Hormosira banksii* from three random sites in each of three source regions (southwest Victoria, eastern Victoria/southern NSW, and Port Phillip Bay) after 14 days of culture at 15, 18, and 21°C showing mean (±*SE*) percentage of embryos with meristematic activity evidenced by apical hair development. Letters above bars indicate Tukey's test results among temperature treatments for each site individually after simple main effects analysis (because of significant *temperature* × *site(region)* interaction in the full model)

**Figure 5:**
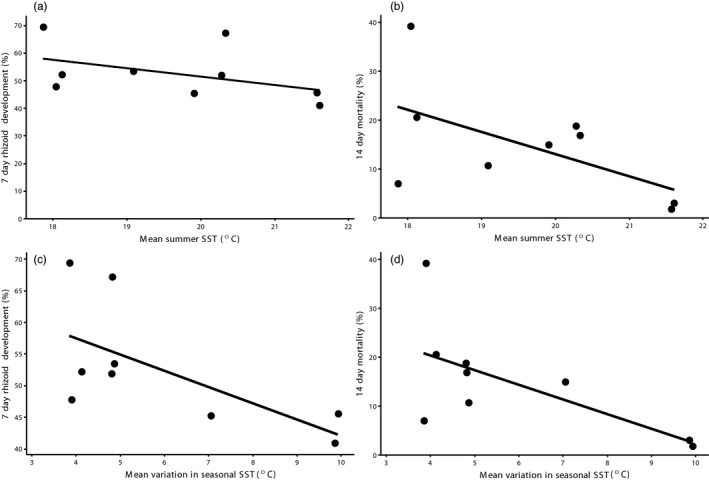
Regression analyses of home site temperature environments and thermal response at 21°C in the common garden experiment. Nonsignificant negative relationships for home site mean summer temperature regressed against (a) 7‐day rhizoid development (*r* = −.45, *p* = .225), and (b) 14‐day mortality (*r* = −.58, *p* = .104). Significant negative relationships between home site mean seasonal variation in sea surface temperature and (c) 7‐day rhizoid development (*r* = −.64, *p* = .007), and (d) 14‐day mortality (*r* = −.65, *p* < .001)

Embryo mortality by day 14 averaged 12.3 ± 0.84% (*N* = 162) but varied among sites within regions with temperature (ANOVA *temperature* × *site(region)* interaction; *F*_(12,135)_ = 3.12; *p* = .001; Figure 3), which was largely driven by significantly higher mortality at 21°C than either 15 or 18°C for one site (Backyards) in the Southwest (Tukey's HSD, *Bonferroni*‐*p* = .017 & .004, respectively; Figure 3). Of the embryos that survived to day 14, meristematic activity (the presence of apical hairs) among temperature treatments was not consistent among sites within regions (ANOVA *temperature* × *site(region)*; *F*_(12,135)_ = 2.66; *p* = .003; Figure 5), but temperature effects on meristematic activity were consistent among regions (ANOVA *temperature* × *region*; *F*_(4,12)_ = 2.55; *p* = .093). Overall, there were significantly more embryos with apical hairs at day 14 in the 21°C treatment compared with both 18 and 15°C treatments (Tukey's HSD, *p* < .001 for 21 versus 18°C and 21 versus 15°C), but this pattern was only significant for 2 sites in each of the PPB and East regions (Tukey's HSD after SME *temperature*; Figure 4).

#### 3.2.4 Regression analyses and *P*_ST_–*F*_ST_ comparisons

Regression analyses suggest an association between home site temperature environments and thermal response in the common garden experiment. We found a significant negative relationship between home site mean seasonal variation in sea surface temperature and 7‐day rhizoid development (*r* = −.64, *p* = .007) and 14‐day mortality (*r* = −.65, *p* < .001) at 21°C (Figure 5). Nonsignificant negative trends were also noted when home site mean summer SST was regressed against 7‐day rhizoid development (*r* = −.45, *p* = .225) and 14‐day mortality (*r* = −.58, *p* = .104) at 21°C (Figure 5). There was no association between home site mean seasonal variation in sea surface temperature or site mean summer temperature with 7‐day rhizoid length (*r* = .05, *p* = .905; *r* = .05, *p* = .908; respectively), and 14‐day apical hair development (*r* = .18, *p* = .647; *r* = .10, *p* = .801; respectively) at 21°C.

Phenotypic variance (*P*_ST_) estimates were highest for 14‐day mortality (*P*_ST_ = 0.85; 95% CI = 0.77–0.95), intermediate for 14‐day apical hair development (*P*_ST_ = 0.79; 95% CI = 0.71–0.92) and 7‐day rhizoid length (*P*_ST_ = 0.70; 95% CI = 0.61–0.88), and lowest for percentage of mature embryos after 7 days (embryos with >4 cell divisions and rhizoids present; *P*_ST_ = 0.44; 95% CI = 0.29–0.81). While *P*_ST_ for percentage of mature embryos was similar to *F*_ST_, *P*_ST_ measures for the remaining three traits were significantly higher than *F*_ST_ globally (*F*_ST_ = 0.24; 95% CI = 0.20–0.29), between regions (*F*_ST_ = 0.09), and within regions (*F*_ST_ = 0.17).

## 4 DISCUSSION

### 4.1 Population connectivity

Genetic and phenotypic differences among populations may contribute to uneven species responses to climate change; such unevenness may result from the genetic history of populations as well as selection associated with prior thermal history. Understanding the interplay of these factors helps predict thermal responses of species to increasing incidences of acute and chronic thermal stress. Within this context, our study on the dominant intertidal macrophyte in Australasia, *H. banksii*, indicates highly restricted gene flow across much of its contemporary distribution with evidence of strong genetic structuring on all spatial scales examined. These findings are consistent with those reported previously for *H. banksii* over smaller geographic ranges (Bellgrove et al., 2017; Coleman et al., 2011, 2019) and reports of fine‐scale genetic structuring in other marine macrophytes including fucoids (Coleman & Brawley, 2005; Williams & Difiori, 1996). Common garden experiments revealed a heterogeneous spatial distribution of thermally tolerant phenotypes, which may be related to local‐scale thermal adaptation. These findings suggest that the impacts on this important foundation species of warming ocean temperatures under climate change may be uneven. Opportunities for recovery of depleted populations and adaptation in populations vulnerable to thermal stress through natural gene flow may be limited, but interventions such as translocation and assisted migration could assist the recovery or bolster the resilience of existing populations given the existence of relatively tolerant populations across the species range (Marzinelli, Leong, Campbell, Steinberg, & Verges, 2016; Wood et al., 2019).

### 4.2 Heterogeneity in thermal response

Consistent with Clark et al. (2013) and Alestra *et al.* (2015), our study suggests the potential for selection to increase thermal tolerance across the species range. Our common garden experiments indicate strong differences in early development between sites sampled from three geographical regions in response to differences in temperature. High water temperatures (21°C) often led to suppressed embryonic development and increased mortality in individuals from sites from cooler regions, while others from warmer and more variable temperature regions were relatively unaffected. Our correlation analyses indicated negative linear relationships between homesite, sea surface temperatures (mean summer temperature and seasonal variation), and thermal response at 21°C. These findings suggest genetically determined variation in thermal tolerance across broad thermal gradients in *H. banksii*, consistent with results reported for other fucoid and laminarian macrophytes (Bennett et al., 2015; King et al., 2018; Mohring, Wernberg, Wright, Connell, & Russell, 2014; Staehr & Wernberg, 2009).

Our results also indicate site variation within regions in the thermal responses of embryos to temperature stress, reflecting potential adaptation on local spatial scales. Although there are few studies on micro‐scale variation in marine macrophytes, signatures of salinity‐associated adaptation among fucoid (*Fucus serratus*) populations separated by as little as 12 km have been previously demonstrated (Coyer et al., 2011), and reciprocal transplants of genotypes from low to high intertidal zones (10s of meters scale) in another fucoid (*Silvetia compressa*) demonstrated clear home site advantages (Hays, 2007). These findings add to a growing literature supporting the notion that selection processes operate on finer spatial scales than currently assumed in marine ecosystems, with local habitat heterogeneity contributing to genetic adaptation in a range of marine organisms (Babin, Gagnaire, Pavey, & Bernatchez, 2017; Miller et al., 2019; Sherman & Ayre, 2008).

While the majority of studies reporting phenotypic variation across environmental gradients in marine macrophytes have not considered the relative contributions of local adaptation and plasticity (King et al., 2018), our study suggests genetic variation among *H. banksii* populations. This is further supported by *Q*_ST_–*F*_ST_ comparisons which indicate departures of *Q*_ST_ from neutral expectations in three of four thermal response traits. Thermal responses are often affected by environmental conditions (i.e., are largely plastic); for instance, macroalgae often show plastic responses in morphology and physiology to changes in temperature (Flukes et al., 2015; Hurd et al., 2014; Reusch, 2014). However, in our case we provide evidence for adaptive heritable variation that can contribute to phenotypic variation alongside this plasticity. The development of heritable differences among sites may reflect limited gene flow in the species. In the absence of gene flow, populations are expected to evolve phenotypes in response to local selection pressures, helping to produce the patterns observed here. Local adaptation over short distances has been shown to occur in terrestrial plant systems when gene flow patterns are conducive to adaptation (e.g., Buehler et al., 2013; Byars, Papst, & Hoffmann, 2007).

Although we have interpreted differences among sites as reflecting genetic variation, such comparisons should ideally involve additional generations because of the possibility of cross‐generation effects induced by environmental conditions (Schiffer, Hangartner, & Hoffmann, 2013). Such effects can lead to phenotypic changes spanning multiple generations (i.e., transgenerational plasticity), including those due to epigenetic mechanisms (Walsh et al., 2016), in the absence of fixed genetic differences that reflect changes in allele frequencies. Cross‐generation studies will help determine the relative contributions of genetic, environmental, and epigenetic effects to the temperature variation we observed among sites.

We have demonstrated substantial genetic and phenotypic differences among *H. banksii* populations, which may manifest in uneven responses to warming sea surface temperatures. Such differences may help to explain nonuniform responses to thermal stress in other macrophyte species. For example, variable responses to temperature stress have been reported at local and regional scales for a range of kelp species from southern California Bight, Nova Scotia, British Columbia, Australia, and New Zealand following extreme climatic events (Bennett et al., 2015; Carballo et al., 2002; Filbee‐Dexter et al., 2016; Starko et al., 2019; Tegner & Dayton, 1987; Thomsen et al., 2019). Such variation is often explained by heterogeneous habitat features (i.e., exposure, depth, bathymetry, and reef geomorphology) that contribute to different thermal environments at local and regional scales (Ierodiaconou et al., 2018). However, habitat differences are also expected to contribute to genetic and phenotypic differences influencing the climate niche of local populations due to selection, particularly for species with low levels of dispersal and strong genetic structuring (King et al., 2018; Miller et al., 2019). This highlights the need for improved evolutionary information on marine macrophytes at the population level to help understand what drives differential responses among populations to thermal stress. Also, this highlights the importance of species distribution and climate niche models that account for population differentiation in order to generate more reliable predictions of species responses to future climates (Martinez et al., 2018; Razgour et al., 2018; Valladares et al., 2014).

Genomic sequencing approaches (i.e., reduced genome representation, whole genome, and transcriptome sequencing) will help in further elucidating patterns of differentiation across sites. Such methods provide unprecedented sensitivity for resolving fine‐scale genetic structure, as well as identifying signatures of selection and putative candidate genes that underlie adaptive differences between species populations (Jordan et al., 2017; Miller et al., 2019). Unfortunately, this study was restricted to the use of microsatellite markers due to DNA not being of sufficient quality for high‐throughput sequencing methods. However, the 10 polymorphic loci used in this study sufficiently resolved patterns of population genetic structure, and microsatellite markers have proven effective for such analyses in other marine macrophyte systems (Reynolds et al., 2017; Wernberg et al., 2018).

### 4.3 Assisted migration and adaptation

Our findings have implications for predicting the recovery potential of depleted *H. banksii* in areas affected by coastal development, urbanization, and thermal stress (Bellgrove et al., 2010; Keough & Quinn, 1998). The apparent lack of gene flow among sites within regions suggests opportunities for natural recolonization following local mortality are low (Coleman et al., 2008). Instead, interventions such as translocation and reseeding may be needed to assist recovery (Bellgrove et al., 2010; Campbell, Marzinelli, Verges, Coleman, & Steinberg, 2014). Moreover, strong population genetic structuring in *H. banksii* also suggests that gene flow is unlikely to assist populations in adapting to warming sea surface temperatures via the migration of thermally adapted genotypes (Alestra & Schiel, 2015; Clark et al., 2013). Instead, a lack of gene flow will necessitate in situ adaptation. While empirical studies suggest that maladaptation can be mitigated in the presence of weak gene flow across environmental gradients (Hoffmann & Sgro, 2011; Sgro et al., 2011), our results suggest that little to no migration is occurring between populations separated by 10s of kilometers. Consequently, the future resilience of local populations to rising sea surface temperatures will depend on standing genetic variation and plasticity, in the absence of any intervention.

While our study suggests that thermally resistant genotypes can occur, selection may struggle to keep pace with a rapidly shifting climatic envelope and increasingly frequent extreme temperature events (Wernberg, Bettignies, et al., 2016). This is particularly pertinent for macrophyte species in climate change hot spots such as southwestern and southeastern Australia. For locally adapted species with limited dispersal ability such as *H. banksii*, adaptive management strategies might be needed. These could include assisted migration of thermally adapted genotypes to populations showing signs of climate stress. Such approaches are being widely advocated as a tool for “climate proofing” threatened marine and terrestrial animal and plant communities (Aitken & Whitlock, 2013; Prober et al., 2015; Sgro et al., 2011). For species showing genetically determined clinal variation in thermal response, such approaches might involve the translocation of genotypes from warm environments into cooler areas (Bansal, Harrington, Gould, St, & B., 2015; Schueler et al., 2013). However, alternative and more tailored approaches may be needed for species such as *H. banksii* where thermal adaptations across the species range are heterogeneous. In such cases, assisted migration strategies may require composite provenancing approaches, involving mixed genotypes from multiple source populations (Broadhurst et al., 2008; Prober et al., 2015; Weeks et al., 2011). This can help to broaden the genetic basis of introduced genotypes, providing at least some genetic variants that are preadapted to warmer oceanographic conditions.

## CONFLICT OF INTEREST

None declared.

## AUTHOR CONTRIBUTIONS

This project was conceived by A.D.M, A.B, M.A.C, and C.D.H.S, with the population genetic data collected by A.D.M, A.B, J.C, and M.A.C, and analysis led by A.D.M. Common garden experiments were led by A.D.M, A.B, and A.A.H; the collection of phenotypic data was assisted by R.C and Z.N, with A.D.M, A.B, A.A.H, M.A.C, and C.D.H.S contributing to data analyses. Writing of the manuscript was led by A.D.M. with assistance from all authors.

## Data Availability

Genetic and phenotypic data will be made publicly available in the DRYAD: ://doi.org/10.5061/dryad.g4f4qrfmb (Miller, 2019).
