# Supplementary material for: Local thermal adaptation and limited gene flow constrain future climate responses of a marine ecosystem engineer
Source: Evol Appl. 2020 Jan 25;13(5):918–34. doi: 10.1111/eva.12909 (PMC7232764; doi:10.1111/eva.12909)
Supplement: Supplementary file 1 [file EVA-13-918-s001.docx]

**SUPPLEMENTARY MATERIAL**

**TABLE S1** Statistics for 26 *Hormosira banksii* collection sites screened with 10 microsatellite loci. Mean values averaged across loci for number of alleles (*a*), expected (*H*_E_) and observed (*H*_O_) heterozygosity, Hardy–Weinberg equilibrium P-values (HWE), and inbreeding coefficients (*F*_IS_). Statistical significance (⍺ = 0.05) after correction for multiple comparisons is indicated by bold text. Migration = the fraction of individuals in the population that are migrants derived from another population per generation (source population code provided).

| **Code** | ***n*** | ***a*** | ***r*** | ***H*_E_** | ***H*_O_** | **HWE** | ***F*_IS_** | **Migration** |
| --- | --- | --- | --- | --- | --- | --- | --- | --- |
| MCM | 24 | 2.38 | 2.22 | 0.37 | 0.31 | 0.11 | 0.03 | 0% |
| TER | 20 | 2.25 | 2.16 | 0.32 | 0.35 | 0.14 | -0.10 | 0% |
| LOR | 24 | 2.63 | 2.49 | 0.43 | 0.43 | 0.26 | 0.00 | 0% |
| SUT | 23 | 2.63 | 2.49 | 0.37 | 0.40 | 0.42 | -0.07 | 5% (LOR) |
| DAL | 22 | 3.25 | 2.94 | 0.49 | 0.44 | 0.03 | 0.06 | 0% |
| MYS | 22 | 3.00 | 2.47 | 0.36 | 0.38 | 0.99 | 0.00 | 0% |
| CPA | 21 | 3.38 | 2.94 | 0.51 | 0.45 | 0.03 | 0.12 | 0% |
| WKV | 20 | 2.38 | 2.36 | 0.43 | 0.35 | 0.56 | 0.00 | 0% |
| FLW | 24 | 2.75 | 2.61 | 0.50 | 0.40 | **<0.01** | **0.21** | 0% |
| LOB | 22 | 3.00 | 2.73 | 0.51 | 0.43 | **<0.01** | **0.15** | 0% |
| BAK | 22 | 2.63 | 2.42 | 0.45 | 0.47 | 0.07 | -0.06 | 0% |
| OIG | 23 | 2.75 | 2.37 | 0.4 | 0.41 | 0.11 | -0.03 | 0% |
| ALD | 15 | 3.25 | 2.94 | 0.52 | 0.45 | 0.03 | 0.15 | 9% (WIL) |
| WIL | 21 | 3.5 | 3.00 | 0.52 | 0.48 | 0.17 | 0.07 | 11% (ALD) |
| GLE | 23 | 2.75 | 2.58 | 0.47 | 0.42 | 0.03 | -0.01 | 0% |
| STN | 23 | 3.13 | 2.57 | 0.42 | 0.45 | 0.76 | -0.08 | 0% |
| POR | 22 | 3.00 | 2.80 | 0.45 | 0.34 | **<0.01** | **0.26** | 0% |
| PTA | 22 | 2.88 | 2.58 | 0.43 | 0.38 | 0.28 | 0.00 | 0% |

**FIGURE S1** Regression analysis for the *Hormosira* *banksii* microsatellite dataset, linearized *F*_ST_ against the natural log of the pairwise oceanic distance (kilometers). In clockwise direction, regression analyses performed on: 1) all sample sites (except Western Australia and New Zealand), 2) Sites from New South Wales only, 3) Sites from Victoria only, 4) Sites from South Australia only. Scores for the accompanying Mantel tests

*r =* 0.35 (*P* < 0.05), *r =* 0.07 (*P* > 0.05), *r =* 0.35 (P < 0.05), *r =* 0.265 (P > 0.05), respectively.


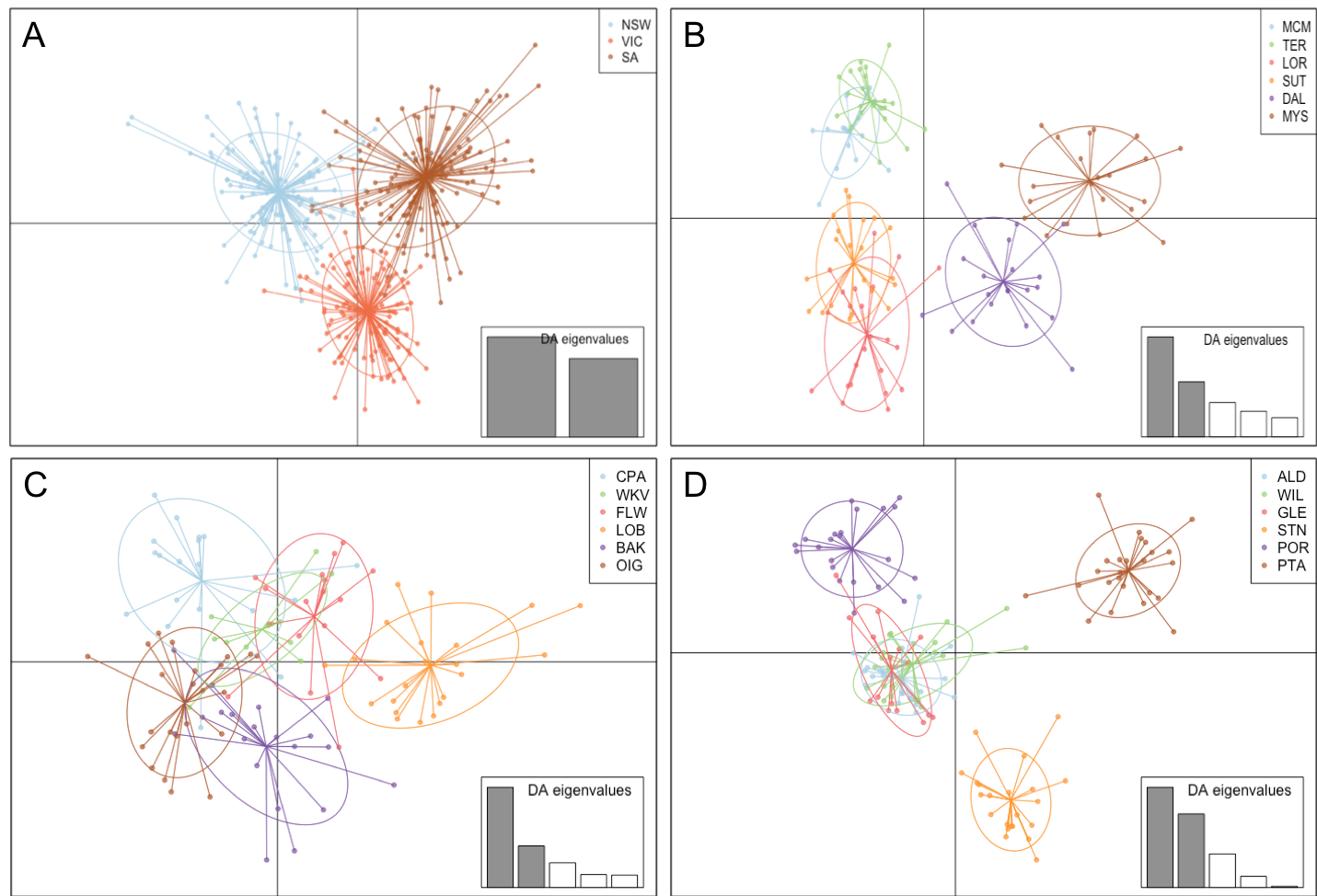


**FIGURE S2** Discriminant Analysis of Principle Components based on microsatellite allele frequencies; A) including individuals from all 26 sites, B) including individuals from NSW sites only, C) including individuals from Victorian sites only, D) including individuals from South Australian sites only.

**
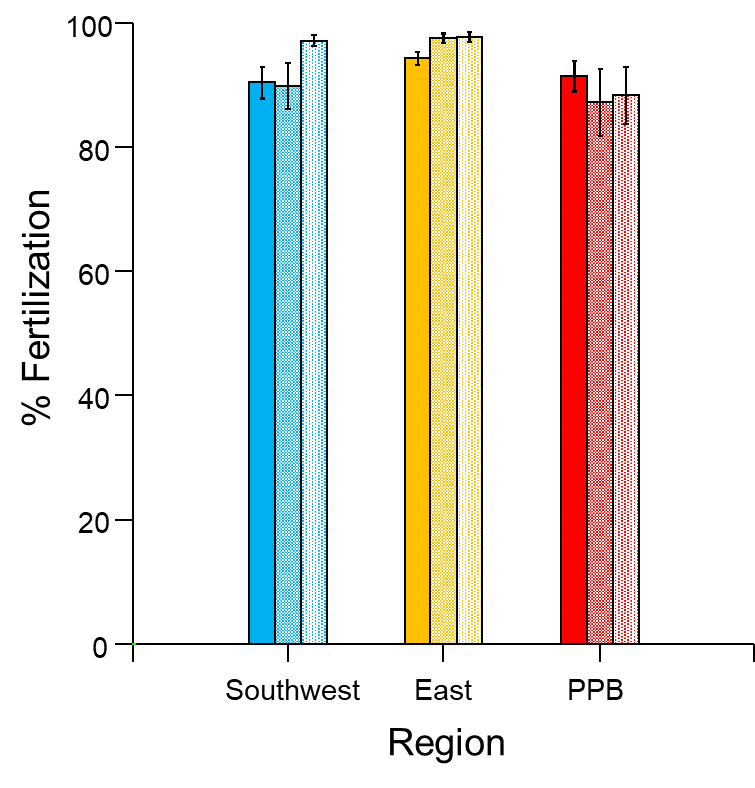
**

**FIGURE S3**. Fertilization success of *Hormosira banksii* from three random sites in each of three source regions (southwest Victoria, eastern Victoria/southern NSW and Port Phillip Bay) prior to allocation to temperature treatments showing mean (± SE) percentage of fertilised zygotes.
